# Walking Speed, Cognitive Function, and Dementia Risk in the English Longitudinal Study of Ageing

**DOI:** 10.1111/jgs.15312

**Published:** 2018-03-06

**Authors:** Ruth A. Hackett, Hilary Davies‐Kershaw, Dorina Cadar, Martin Orrell, Andrew Steptoe

**Affiliations:** ^1^ Department of Behavioural Science and Health University College London London United Kingdom; ^2^ School of Health Sciences University of Surrey Guildford United Kingdom; ^3^ Institute of Mental Health University of Nottingham Nottingham United Kingdom

**Keywords:** gait speed, cognition, dementia

## Abstract

**Objectives:**

To determine the relationships between walking speed, cognitive function, and the interaction between changes in these measures and dementia risk.

**Design:**

Longitudinal observational study.

**Setting:**

English Longitudinal Study of Ageing.

**Participants:**

Individuals aged 60 and older (N=3,932).

**Measurements:**

Walking speed and cognition were assessed at Waves 1 (2002–03) and 2 (2004–05) of the English Longitudinal Study of Ageing. New dementia cases were assessed from Wave 3 (2006–07) to Wave 7 (2014–15). The associations were modelled using Cox proportional hazards regression.

**Results:**

Participants with faster baseline walking speeds were at lower risk of developing dementia (hazard ratio (HR)=0.36, 95% confidence interval (CI)=0.22–0.60). Those with a greater decline in walking speed from Wave 1 to 2 were at greater risk of developing dementia (HR=1.23, 95% CI=1.03–1.47). Participants with better baseline cognition (HR=0.42, 95% CI=0.34–0.54) were at lower risk of developing dementia. Those with a greater decline in cognition from Wave 1 to 2 were at greater risk of developing dementia (HR=1.78, 95% CI=1.53–2.06). Change in walking speed and change in cognition did not have an interactive effect on dementia risk (HR=1.01, 95% CI=0.88–1.17).

**Conclusion:**

In this community‐dwelling sample of English adults, those with slower walking speeds and a greater decline in speed over time were at greater risk of developing dementia independent of changes in cognition. Further research is required to understand the mechanisms that may drive these associations.

## Abstract 2

See related editorial by https://doi.org/https://doi.org/10.1111/jgs.15368.

In 2015, an estimated 46.8 million people worldwide had dementia.^1^ Dementia is a contributor to disability and life years lost in older individuals.^2^ There is no cure for dementia, so identifying potential risk factors may reveal opportunities for prevention. One area of interest is whether physical function is related to dementia onset, because declines in physical and cognitive functioning are indicators of aging, and gait disorders increase with age^3^ and are associated with incident dementia.^4^

Walking speed is easier to assess than other gait parameters. Slow walking speed is associated with negative outcomes in older individuals.^5^, ^6^, ^7^ Individuals with cognitive impairment and dementia walk more slowly than individuals without these conditions 8 Furthermore, meta‐analytical evidence indicates that slow walking speed is a predictor of dementia.^4^, ^9^ Change in walking speed in relation to dementia risk has been less well researched than current walking speed. But in 3,663 French adults, those with a steeper decline in walking speed were at greater risk of dementia than those with a slower decline.^10^ Similar findings have been reported in Swedish and Japanese samples.^11^, ^12^

Dementia develops slowly and can be preceded by years of decline in cognitive functioning.^13^ Cognition and physical function influence one another in a complex manner.^14^ There is some evidence that the association between cognition and walking speed is bidirectional,^15^ although most studies find that slow walking is a predictor of decline in cognition but not vice versa.^16^, ^17^

The relationship between changes in cognition and walking speed has been assessed in several studies, but the results are equivocal.^18^, ^19^, ^20^ Findings from a sample of 762 participants in the MacArthur Studies of Successful Aging showed that cognition and walking speed declined in tandem over a 7‐year period.^18^ A limited association was also detected in a Tasmanian sample, in which a decline in executive function (but no other domains) was associated with a decrease in walking speed.^19^ An analysis from the Women's Health Initiative Memory Study failed to detect any association.^20^

Overall, it appears that slow walking speed^9^ is associated with greater dementia risk, with more‐limited evidence that faster decline in walking speed is also relevant.^10^, ^11^, ^12^ The evidence is mixed as to whether changes in cognition are associated with changes in walking speed,^18^, ^19^, ^20^ and these associations have not been examined in relation to dementia risk. To address these questions, we evaluated whether walking speed and change in walking speed were predictive of dementia in a sample of 3,932 English adults. We also evaluated whether changes in cognition and walking speed have an interactive effect on dementia risk.

## Methods

### Study Population

Our data were from the English Longitudinal Study of Ageing (ELSA): a representative study of community‐dwelling adults aged 50 and older in England.^21^ Data collection began in 2002–03, with follow‐up every 2 years. We used data from Waves 1 (2002–03) to 7 (2014–15). All participants provided informed consent. Ethical approval was obtained from the National Research Ethics Committee.

### Dementia

The outcome was incident dementia from Wave 3 (2006–07) to Wave 7 (2014–15). We used 2 information sources to define dementia, as in previous work.^22^, ^23^, ^24^ The primary indicator was self‐reported physician‐diagnosed dementia. The second criterion was applied to participants who were not able to respond directly and was a caregiver's comparison of functional performance with that from 2 years before. We used a 16‐item adapted short‐form version of the Informant Questionnaire on Cognitive Decline in the Elderly (IQCODE)^25^ and defined individuals with an average score of 3.5 or greater as having dementia, based on previous work suggesting high sensitivity and specificity at this cut‐point.^26^ There were 289 incident cases of dementia between Waves 3 and 7, 240 of which were based on physician‐diagnosed dementia and 49 on IQCODE scores.

### Walking Speed

Walking speed was assessed in participants aged 60 and older. Participants were asked to walk a distance of 8 feet (2.43 m) from a standing start on even ground at their usual pace, and the time taken was recorded. The average time of two walks was calculated. We used walking speed at Wave 1 (2002–03) as a predictor of dementia.

### Cognitive Function

We aggregated information from 4 cognitive tests (memory (immediate and delayed), time orientation, verbal fluency, processing speed) to obtain cognitive function scores at Waves 1 and 2. To compute an overall score, we transformed each of the measures into a z score and derived average total scores in 2002–03 and 2004–05. More information about these tasks is provided elsewhere.^27^

### Covariates

We classified age into 3 categories (60–69, 60–79, ≥80). Socioeconomic status was defined using deciles of nonpension wealth (1=low, 10=high). We divided education into 3 categories (<junior high school, high school, university). Mobility impairment at baseline was ascertained by asking participants whether they had difficulty with one or more common arm and leg functions (e.g., getting in or out of bed). Activity of daily living (ADL) impairment was indexed by asking participants whether they had difficulty with 6 activities (e.g., dressing, including putting on shoes and socks,). Physician diagnoses of coronary heart disease, stroke, diabetes, cancer and hypertension were entered as binary (yes/no) variables, because these conditions may affect dementia risk. Because depression is associated with dementia onset, we included the 8‐item Center for Epidemiologic Studies Depression Scale (CES‐D) score. Baseline cognitive function was included in all analyses. Baseline walking speed was controlled for in analyses examining change in walking speed as a predictor of future dementia.

### Statistical Analysis

We compared characteristics at Wave 1 of participants who developed dementia with characteristics of those who did not using logistic regression and univariate analysis of variance. Because age is an important factor in dementia risk, we controlled for age. We used Cox proportional hazards regression to model the association between walking speed at Wave 1 (2002–03) and cumulative dementia from Wave 3 (2006–07) to Wave 7 (2014–15). Participants who had dementia at Waves 1 and 2 were excluded. If the precise date of dementia diagnosis was unknown, we used the midpoint date between the waves of data collection. We censored individuals who dropped out of the study or died. We used the last ELSA interview date as the censor date. We calculated change scores in walking speed and cognition by subtracting values in 2004–05 (Wave 2) from values in 2002–03 (Wave 1). We then computed an interaction term between changes in walking speed and cognitive function. Preliminary analyses removing those who died did not change the pattern of results. We conducted 3 sensitivity analyses. First, we modelled the association between walking speed at Wave 2 (2004–05) and cumulative dementia risk (Waves 3–7). Second, we used only physician‐diagnosed dementia as the outcome. Third, we conducted the analyses including new dementia cases from Wave 4 to 7 (2008–2015) rather than from Wave 3 to 7. Results are presented as hazard ratios (HRs) and 95% confidence intervals (95% CI). All analyses were conducted using SPSS version 24 (IBM Corp., Armonk, NY).

## Results

### Participant Characteristics

We compared the characteristics of those who developed dementia (n= 289) with the characteristics of those who did not (n=3,643). Those who developed dementia were significantly older on average, were less wealthy, had poorer cognition and mobility and significantly slower walking speed, and were more likely to have had a stroke or depressive symptoms than those who did not (Table 1).

**Table 1:** Participant Characteristics (2002–04) According to Dementia Status (2006–15)

Characteristic	Dementia, n = 289	No Dementia, n = 3,643	P‐Value
Age, n (%)			<.001
60–69	63 (21.8)	2,016 (55.3)	
70–79	135 (46.7)	1,248 (34.3)	
≥80	91 (31.5)	379 (10.4)	
Male, n (%)	110 (38.2)	1,621 (44.5)	.06
Wealth, decile, mean	5.34 ± 0.17	5.85 ± 0.05	.004
Education, n (%)			.70
<Junior high school	125 (43.2)	1,512 (41.5)	
High school	99 (34.3)	1,293 (35.5)	
University	65 (22.5)	838 (23)	
Walking speed, m/s, mean ± SD	0.78 ± 0.02	0.87 ± 0.01	<.001
Cognition, mean ± SD (range −2.97 to 4.02, Z score)	–0.32 ± 0.04	–0.06 ± 0.01	<.001
Mobility impairment, mean ± SD (range 0–10)^a^	2.44 ± 0.14	1.95 ± 0.04	.001
Activity of daily living difficulty, mean ± SD (range 0–6)^b^	0.41 ± 0.04	0.32 ± 0.01	.05
Coronary heart disease, n (%)	50 (17.4)	532 (14.6)	.16
Stroke, n (%)	22 (7.5)	149 (4.1)	.02
Diabetes, n (%)	25 (8.8)	270 (7.4)	.26
Hypertension, n (%)	132 (45.6)	1,537 (42.2)	.16
Cancer, n (%)	16 (5.6)	189 (5.2)	.67
Center for Epidemiologic Studies Depression Scale score, mean ± SD (range 0–8)	1.83 ± 0.11	1.38 ± 0.03	<.001

All analyses are age‐adjusted.

a Difficulty with ≥1 common arm and leg functions (range 0–10).

b Whether participants had difficulties with 6 daily activities (range 0–6).

SD = standard deviation.

### Baseline Walking Speed as a Predictor of Dementia

Walking speed at Wave 1 was a predictor of development of dementia, with those with faster walker speeds being less likely to develop dementia (HR=0.36, 95% CI=0.22–0.60) during follow‐up (Table 2). This association was robust to adjustment for covariates. Cognitive function at baseline was also an independent predictor of development of dementia (HR=0.42, 95% CI=0.33–0.52), with those with better cognitive function being less likely to develop dementia.

**Table 2:** Hazard Ratio of Walking Speed (2002–03) and Dementia Incidence (2006–15)

Factor	Adjusted HR (95% Confidence Interval)	P‐Value
Walking speed, m/s	0.36 (0.22–0.60)	<.001
Cognition^a^	0.42 (0.33–0.52)	<.001
Sex	1.09 (0.84–1.42)	.48
Age (reference 60–69)		
70–79	2.91 (2.14–3.94)	<.001
≥80	6.68 (4.70–9.49)	<.001
Wealth, decile	0.96 (0.92–1.01)	.09
Education (reference <junior high school)		
High school	1.01 (0.76–1.33)	.960
University	1.24 (0.87–1.77)	.242
Mobility impairment^b^	0.98 (0.92–1.05)	.596
Activity of daily living difficulty	1.03 (0.87–1.21)	.762
Coronary heart disease	1.12 (0.83–1.52)	.446
Stroke	1.26 (0.82–1.93)	.295
Diabetes mellitus	1.11 (0.74–1.65)	.614
Hypertension	1.06 (0.84–1.34)	.626
Cancer	1.03 (0.63–1.70)	.894
Depression	1.06 (0.99–1.13)	.087

a 1–standard deviation increment in cognition associated with adjusted hazard ratio (HR).

b Difficulty with ≥1 common arm and leg functions (range 0–10).

### Changes in Walking Speed and Cognition as Predictors of Dementia

Walking speed decreased on average from 0.86 to 0.85 m/s between Waves 1 and 2. Change in walking speed was a significant predictor of dementia (HR=1.23, 95% CI=1.03–1.47), with those who had a greater decrease in walking speed from Wave 1 to Wave 2 having a greater risk of developing dementia independent of covariates and walking speed in Wave 1 (Table 3). Change in cognition was also a predictor, with participants with a greater significant decline in cognitive function between Waves 1 and 2 being at greater risk of developing dementia (HR=1.78, 95% CI=1.53–2.06), but the interaction between walking speed and cognitive function was not a significant predictor of dementia (HR=1.01, 95% CI=0.88–1.17).

**Table 3:** Hazard Ratio of Change in Walking Speed and Cognition (from 2002–03 to 2004–05) and Dementia Incidence (2006–15)

Factor	Adjusted HR (95% Confidence Interval)	P‐Value
Change in walking speed, m/s	1.23 (1.03–1.47)	.02
Change in cognition, z score^a^	1.78 (1.53–2.06)	< .001
Walking by cognition interaction	1.01 (0.88–1.17)	.88
Baseline walking speed, m/s^b^	0.28 (0.14–0.57)	< .001
Baseline cognition^b^	0.27 (0.21–0.35)	< .001
Female	1.19 (0.89–1.56)	.23
Age (reference 60–69)		
70–79	3.03 (2.17–4.22)	< .001
≥80	6.59 (4.49–9.68)	< .001
Wealth (decile)	0.97 (0.92–1.02)	.28
Education (reference <junior high school)		
High school	0.96 (0.71–1.31)	.80
University	1.44 (0.97–2.13)	.07
Mobility impairment^c^	0.99 (0.92–1.06)	.68
Activity of daily living difficulty	0.99 (0.82–1.20)	.90
Coronary heart disease	1.21 (0.87–1.67)	.26
Stroke	1.04 (0.63–1.70)	.88
Diabetes mellitus	1.18 (0.77–1.81)	.45
Hypertension	0.96 (0.75–1.24)	.77
Cancer	1.08 (0.62–1.90)	.79
Depression	1.03 (0.96–1.10)	.48

a 1–standard deviation increment in cognition associated with adjusted hazard ratio (HR).

b 1‐m/s increment in walking speed associated with adjusted HR.

c Difficulty with ≥1 common arm and leg functions (range 0–10).

### Sensitivity Analyses

Three sensitivity analyses were conducted. First, we tested whether walking speed at Wave 2 (2004–05) predicted development of dementia. Participants with faster walking speeds were less likely to develop dementia (HR=0.25) between Waves 3 and 7 (2006–2015) (Supplementary Table S1).

Second, we only considered new events from Wave 4 to 7, omitting any that occurred within 2 years of Wave 2. This reduced the number of cases from 289 to 225 (Supplementary Table S2), but walking speed at Wave 1 remained a predictor of dementia (HR=0.33). Similarly, those with poorer cognition at Wave 1 (HR=0.29) and those who had a greater decline in cognitive function between Waves 1 and 2 (HR=1.69) remained more likely to develop dementia. The effect size for changes in walking speed was similar to that in the full analysis, but the association was no longer significant because of the smaller number of cases.

For the final sensitivity analysis, we excluded participants who were diagnosed using the IQCODE and limited the analysis to those with physician diagnoses. This reduced the number of cases from 289 to 240 (Supplementary Table S3). Walking speed remained a predictor of dementia (HR=0.36). The findings for cognition also remained for baseline cognition (HR=0.26) and change in cognitive function (HR=1.83), but similar to the other sensitivity analysis, change in walking speed no longer predicted development of dementia.

## Discussion

In this sample of 3,932 older adults, those with a slower walking speed were at greater risk of developing dementia. Individuals who experienced a greater decline in walking speed were also at greater risk. Participants with poorer cognition at baseline and those who experienced greater decline in cognition were also more likely to be diagnosed with dementia, although change in walking speed and change in cognition did not have an interactive effect on development of dementia.

Our finding that slower walking speed is a predictor of subsequent dementia is in line with previous research.^4^, ^9^ In a meta‐analysis of 10 prospective studies in this area, 9 were conducted in the United States and one in Sweden.^9^ Our study adds to this literature by demonstrating an association between walking speed and dementia in an English sample. To the best of our knowledge, this is also the largest sample used to address this question. The association remained significant in our sensitivity analyses, demonstrating the robustness of this finding.

The association between change in walking speed and dementia risk has not been investigated as much. In the current study, those with greater decline in walking speed over 2 measurement periods were more likely to develop dementia. This finding is in agreement with research in Swedish, French, and Japanese cohorts.^10^, ^11^, ^12^ The relationship between change in walking speed and dementia remained after controlling for numerous covariates, including baseline walking speed. This suggests that, regardless of initial walking speed, a marked decrease over a relatively short period (2 years) may be an indicator of greater dementia risk.

We were interested in how cognition and walking speed might influence one another in affecting dementia risk. First, we found that individuals who had poorer baseline cognition were more likely to develop dementia. Second, those with greater decline in cognitive function between Waves 1 and 2 were at higher risk of dementia over follow‐up. Finally, we investigated whether changes in cognition and walking speed interacted to affect risk of development of dementia, but we found no evidence to support this.

Initial cognitive function and decline in cognitive ability are associated with greater risk of subsequent dementia,^13^ but understanding of the interplay between changes in walking speed and cognition is limited. Previous research attempting to ascertain whether these functions decline in parallel has been inconsistent, with one study suggesting that these functions decline in tandem,^18^ another finding an association but only for one component of cognition (executive function),^19^ and another finding no association at all.^20^

The present study extends this research by investigating whether changes in cognition and in walking speed interact to predict future dementia. Our findings suggest that declining walking speed and declining cognitive function are independent predictors of dementia but that these factors do not work synergistically. Cross‐sectional evidence suggests that gait speed maps onto the stage of cognitive impairment; with the fastest speeds reported in individuals with mild cognitive impairment and the slowest speeds reported in those with advanced dementia.^28^ It may be that this interplay between cognition and walking speed emerges only at the stage of clinically significant impairment rather than when individuals are still cognitively healthy.

This observational study provides information on the chronological, but not the causal, relationship between walking speed and dementia. Furthermore, reverse causality might be a factor. There is weak evidence from some^15^, ^16^ but not all studies^17^ that cognitive function may predict walking speed and that walking speed might influence later cognitive status. In our sensitivity analysis excluding cases that occurred in the first 6 years after the walking speed assessment, the association between walking speed and subsequent dementia remained. This addresses the notion that undetected dementia cases affected walking speed and adds weight to the temporal sequence that gait slowing precedes dementia onset.

With regard to the mechanisms linking walking speed with future dementia, several possibilities could help explain our findings. It is thought that walking and cognition rely on similar brain regions, predominately in the prefrontal cortex.^29^, ^30^ Gait is a complex process in which the locomotor systems receive input from the basal ganglia, motor cortex, and cerebellum.^30^ Although this process is largely automatic, walking relies on sensory feedback and high‐order cognitive control.^29^, ^30^

Neurodegeneration is a possible underlying mechanism linking declines in physical and cognitive function, with changes in subcortical white matter^31^ and cortical gray matter volumes^31^ associated with slower gait speeds. Vascular risk factors may also contribute to the link between gait speed decline and dementia through a similar pathway, because micro damage to the vessels of the prefrontal cortex and lesions (e.g., from stroke) is associated with white matter changes,^32^ but our analyses were robust to adjustment for vascular risk factors and history of cardiometabolic disease.

Another potential mechanism is low‐grade systemic inflammation. High concentrations of inflammatory markers are predictive of new‐onset dementia^33^ and have also been implicated in mobility impairment.^34^ Neuroinflammation is thought to lead to impaired neuroplasticity in the brain areas controlling motor and cognitive function.^30^ Furthermore, walking speed relies on muscle strength,^35^ and muscle loss has been linked with deleterious inflammatory processes.^36^

These findings may have implications for efforts to delay dementia onset. The effect of exercise interventions on cognitive function and dementia is unclear.^37^, ^38^ Nonetheless, a meta‐analysis of 42 studies examining the effects of 3 exercise interventions on walking speed^39^ suggested that exercise can lead to increases in walking speed of up to 9.3%. Exercise may also improve cognitive function. A 2017 meta‐analysis of 36 randomized control trials found that various types of exercise had a beneficial effect on cognition, regardless of baseline cognitive status.^40^

We analyzed data from a large representative sample of English adults using an objective test of walking speed and were able to confirm associations between walking speed and dementia risk after adjusting for multiple confounders, such as mobility impairment and cardiometabolic disease. Dementia was primarily identified according to self‐reported physician diagnoses, which resulted in fewer cases than would be expected based on population estimates.^41^ When this was supplemented by including diagnoses based on the IQCODE, the incidence of dementia was in line with U.K. estimates.^41^ Although it is possible that we missed cases, given the consistency of our findings with earlier studies,^4^, ^9^, ^10^, ^11^, ^12^ it is unlikely that misclassification bias accounted for our results. Furthermore, self‐reported diagnoses of other conditions are noted to correspond closely with physician diagnoses, even in individuals with cognitive impairment.^42^

The average decrease in walking speed between Waves 1 and 2 was small and may not be clinically significant. Although the findings of our study suggest that individuals with larger declines in speed are more likely to develop dementia. Some aspects of cognition are thought to be more strongly associated with physical functioning than others.^11^, ^15^ However, this study did not investigate associations between walking speed and different aspects of cognition, meaning that potential interactions before dementia onset may have been missed.

Overall, this study suggests that individuals with slower walking speeds and those who have a greater decline in walking speed over time are at greater risk of dementia. Changes in walking speed and in cognition did not interact to affect development of dementia, indicating that they operate through independent pathways. Further research is required to understand the causal mechanisms underlying these associations and to determine whether increases in walking speed reduce dementia risk.

## Supporting information


**Table S1:** Cox proportional hazards regression of the incidence of dementia from Waves 3–7 (2006–2015) on walking speed at Wave 2 (2004–2005)
**Table S2:** Cox proportional hazards regression of the incidence of dementia from Waves 4–7 (2008–2015) on walking speed & cognitive function between Waves 1 and 2 (2002–2005)
**Table S3:** Cox proportional hazards regression of the incidence of doctor diagnosed dementia from Waves 3–7 (2006–2015) on change in walking speed & cognitive function between Waves 1 and 2 (2002–2005)
