# Supplementary material for: Walking Speed, Cognitive Function, and Dementia Risk in the English Longitudinal Study of Ageing
Source: J Am Geriatr Soc. 2018 Mar 6;66(9):1670–5. doi: 10.1111/jgs.15312 (PMC6127007; doi:10.1111/jgs.15312)
Supplement: Supplementary file 1 — Table S1: Cox proportional hazards regression of the incidence of dementia from Waves 3–7 (2006–2015) on walking speed at Wave 2 (2004–2005) Table S2: Cox proportional hazards regression of the incidence of dementia from Waves 4–7 (2008–2015) on walking speed & cognitive function between Waves 1 and 2 (2002–2005) Table S3: Cox proportional hazards regression of the incidence of doctor diagnosed dementia from Waves 3–7 (2006–2015) on change in walking speed & cognitive function between Waves 1 and 2 (2002–2005) [file JGS-66-1670-s001.docx]

**Supplementary Table 1: Cox proportional hazards regression of the incidence of dementia from waves 3-7 (2006-2015) on walking speed at wave 2 (2004-2005)**

|  | **Adjusted HR**  **(95% C.I.)** | **p value** |
| --- | --- | --- |
| Walking speed (m/s)* | 0.25 (0.13 – 0.47) | < 0.001 |
| Cognitive function† | 0.42 (0.34 – 0.54) | < 0.001 |
| Sex^1^ | 1.09 (0.83 – 1.45) | = 0.516 |
| Age 60-69 years  70-79  ≥80 | 1  2.92 (2.11 – 4.07)  7.26 (4.97 – 10.61) | < 0.001  < 0.001 |
| Wealth (decile) ^2^ | 0.97 (0.93 – 1.03) | = 0.383 |
| Education: Lower^3^  Intermediate  Higher | 1  0.86 (0.64 – 1.17)  1.17 (0.80 – 1.72) | = 0.346  = 0.417 |
| Mobility impairments^4^  ADL^5^ | 0.97 (0.91 – 1.05)  1.01 (0.84 – 1.22) | = 0.491  = 0.896 |
|  |  |  |
| CHD^6^ | 1.19 (0.86 – 1.65) | = 0.281 |
| Stroke^6^ | 1.14 (0.69 – 1.86) | = 0.608 |
| Diabetes^6^ | 1.17 (0.77 – 1.77) | = 0.472 |
| Hypertension^6^ | 0.95 (0.74 – 1.23) | = 0.688 |
| Cancer^6^ | 1.16 (0.66 – 2.04) | = 0.602 |
| Depression | 1.06 (0.99 – 1.14) | = 0.105 |
|  |  |  |

ADL= Activities of daily living; CHD= Coronary Heart Disease; HR= Hazard Ratio

*1 m/s increment in walking speed associated with adjusted HR

† 1 SD increment in cognition associated with adjusted HR

Note: ^1^ Male is the reference group; ^2^A higher number represents greater wealth

^3^ Lower = no qualifications; intermediate = junior high & high school; higher = university

^4^ Whether participants had difficulties with ≥1 common arm & leg functions (range 0-10)

^5^ Whether participants had difficulties with 6 daily activities (range 0-6)

^6^No illness is the reference group

**Supplementary Table 2: Cox proportional hazards regression of the incidence of dementia from waves 4-7 (2008-2015) on walking speed & cognitive function between waves 1 and 2 (2002-2005)**

|  | **Adjusted HR**  **(95% C.I.)** | **p value** |
| --- | --- | --- |
| Change in walking speed (m/s) | 1.20 (0.98 – 1.45) | = 0.068 |
| Change in cognition (z score) | 1.69 (1.42 – 2.01) | < 0.001 |
| Walking by cognition interaction | 1.04 (0.86 – 1.22) | = 0.689 |
| Walking speed wave 1 (m/s)* | 0.33 (0.15 – 0.73) | = 0.007 |
| Cognition function wave 1† | 0.29 (0.22 – 0.39) | < 0.001 |
|  |  |  |
| Sex^1^ | 1.14 (0.84 – 1.55) | = 0.415 |
| Age 60-69 years  70-79  ≥80 | 1  3.00 (2.10 – 4.29)  6.89 (4.52 – 10.51) | < 0.001  < 0.001 |
| Wealth (decile) ^2^ | 0.97 (0.91 – 1.03) | = 0.258 |
| Education: Lower^3^  Intermediate  Higher | 1  1.01 (0.72 – 1.41)  1.45 (0.94 – 2.24) | = 0.976  = 0.096 |
| Mobility impairments^4^  ADL^5^ | 0.99 (0.92 – 1.08)  1.01 (0.81 – 1.26) | = 0.918  = 0.933 |
|  |  |  |
| CHD^6^ | 1.23 (0.85 – 1.78) | = 0.275 |
| Stroke^6^ | 0.96 (0.54 – 1.72) | = 0.907 |
| Diabetes^6^ | 1.20 (0.74 – 1.95) | = 0.450 |
| Hypertension^6^ | 0.94 (0.71 – 1.25) | = 0.670 |
| Cancer^6^ | 0.82 (0.40 – 1.68) | = 0.594 |
| Depression | 1.05 (0.96 – 1.13) | = 0.267 |
|  |  |  |

ADL= Activities of daily living; CHD= Coronary Heart Disease; HR= Hazard Ratio

*1 m/s increment in walking speed associated with adjusted HR

† 1 SD increment in cognition associated with adjusted HR

Note: ^1^ Male is the reference group; ^2^A higher number represents greater wealth

^3^ Lower = no qualifications; intermediate = junior high & high school; higher = university

^4^ Whether participants had difficulties with ≥1 common arm & leg functions (range 0-10)

^5^ Whether participants had difficulties with 6 daily activities (range 0-6)

^6^No illness is the reference group

**Supplementary Table 3: Cox proportional hazards regression of the incidence of doctor diagnosed dementia from waves 3-7 (2006-2015) on change in walking speed & cognitive function between waves 1 and 2 (2002-2005)**

|  | **Adjusted HR**  **(95% C.I.)** | **p value** |
| --- | --- | --- |
| Change in walking speed (m/s) | 1.17 (0.97 – 1.42) | = 0.102 |
| Change in cognition (z score) | 1.83 (1.55 – 2.16) | < 0.001 |
| Walking by cognition interaction | 1.03 (0.88 – 1.21) | = 0.708 |
| Walking speed wave 1 (m/s)* | 0.36 (0.16 – 0.79) | = 0.012 |
| Cognition function wave 1† | 0.26 (0.19 – 0.34) | < 0.001 |
|  |  |  |
| Sex^1^ | 1.27 (0.94 – 1.74) | = 0.120 |
| Age 60-69 years  70-79  ≥80 | 1  3.13 (2.17 – 4.51)  7.21 (4.72 – 10.99) | < 0.001  < 0.001 |
| Wealth (decile)^2^ | 0.97 (0.92 – 1.03) | = 0.342 |
| Education: Lower^3^  Intermediate  Higher | 1  0.88 (0.62 – 1.24)  1.43 (0.93 – 2.20) | = 0.463  = 0.100 |
| Mobility impairments^4^  ADL^5^ | 0.99 (0.92 – 1.08)  0.93 (0.74 – 1.17) | = 0.906  = 0.535 |
|  |  |  |
| CHD^6^ | 1.18 (0.82 – 1.69) | = 0.381 |
| Stroke^6^ | 0.97 (0.55 – 1.74) | = 0.940 |
| Diabetes^6^ | 1.19 (0.73 – 1.94) | = 0.490 |
| Hypertension^6^ | 0.90 (0.68 – 1.20) | = 0.477 |
| Cancer^6^ | 1.09 (0.59 – 2.02) | = 0.777 |
| Depression | 1.02 (0.94 – 1.10) | = 0.693 |
|  |  |  |

ADL= Activities of daily living; CHD= Coronary Heart Disease; HR= Hazard Ratio

*1 m/s increment in walking speed associated with adjusted HR

† 1 SD increment in cognition associated with adjusted HR

Note: ^1^ Male is the reference group; ^2^A higher number represents greater wealth

^3^ Lower = no qualifications; intermediate = junior high & high school; higher = university

^4^ Whether participants had difficulties with ≥1 common arm & leg functions (range 0-10)

^5^ Whether participants had difficulties with 6 daily activities (range 0-6)

^6^No illness is the reference group
